# Biologically induced mineralization of dypingite by cyanobacteria from an alkaline wetland near Atlin, British Columbia, Canada

**DOI:** 10.1186/1467-4866-8-13

**Published:** 2007-12-05

**Authors:** Ian M Power, Sasha Wilson, James M Thom, Gregory M Dipple, Gordon Southam

**Affiliations:** 1 Department of Earth Sciences, The University of Western Ontario, London, Ontario N6A 5B7, Canada; 2 Mineral Deposit Research Unit, Department of Earth and Ocean Sciences, The University of British Columbia, Vancouver, British Columbia V6T 1Z4, Canada

## Abstract

**Background:**

This study provides experimental evidence for biologically induced precipitation of magnesium carbonates, specifically dypingite (Mg_5_(CO_3_)_4_(OH)_2_·5H_2_O), by cyanobacteria from an alkaline wetland near Atlin, British Columbia. This wetland is part of a larger hydromagnesite (Mg_5_(CO_3_)_4_(OH)_2_·4H_2_O) playa. Abiotic and biotic processes for magnesium carbonate precipitation in this environment are compared.

**Results:**

Field observations show that evaporation of wetland water produces carbonate films of nesquehonite (MgCO_3_·3H_2_O) on the water surface and crusts on exposed surfaces. In contrast, benthic microbial mats possessing filamentous cyanobacteria (*Lyngbya *sp.) contain platy dypingite (Mg_5_(CO_3_)_4_(OH)_2_·5H_2_O) and aragonite. Bulk carbonates in the benthic mats (δ^13^C avg. = 6.7‰, δ^18^O avg. = 17.2‰) were isotopically distinguishable from abiotically formed nesquehonite (δ^13^C avg. = 9.3‰, δ^18^O avg. = 24.9‰). Field and laboratory experiments, which emulated natural conditions, were conducted to provide insight into the processes for magnesium carbonate precipitation in this environment. Field microcosm experiments included an abiotic control and two microbial systems, one containing ambient wetland water and one amended with nutrients to simulate eutrophic conditions. The abiotic control developed an extensive crust of nesquehonite on its bottom surface during which [Mg^2+^] decreased by 16.7% relative to the starting concentration. In the microbial systems, precipitation occurred within the mats and was not simply due to the capturing of mineral grains settling out of the water column. Magnesium concentrations decreased by 22.2% and 38.7% in the microbial systems, respectively. Laboratory experiments using natural waters from the Atlin site produced rosettes and flakey globular aggregates of dypingite precipitated in association with filamentous cyanobacteria dominated biofilms cultured from the site, whereas the abiotic control again precipitated nesquehonite.

**Conclusion:**

Microbial mats in the Atlin wetland create ideal conditions for biologically induced precipitation of dypingite and have presumably played a significant role in the development of this natural Mg-carbonate playa. This biogeochemical process represents an important link between the biosphere and the inorganic carbon pool.

## 1. Background

The contribution to carbonate precipitation and sedimentation by microorganisms has been substantial since the Archaean. In these ancient and in contemporary systems, cyanobacteria are considered to have played a role in carbonate precipitation within oceans, lakes, springs, caves, and soils [1]. Experimental evidence supports biologically mediated precipitation in a wide-range of environments with various carbonate mineralogies. Thompson and Ferris [2] conducted unique laboratory experiments simulating carbonate precipitation in Fayetteville Green Lake, New York using the unicellular cyanobacterium, *Synechococcus *sp. Other experimental studies linked microbial activity to the precipitation of calcium carbonates [3,4] and dolomite [5-7]. Thompson and Ferris [2] suggested that biologically induced precipitation of magnesium carbonates might occur at pH values > 8.5 in aquatic environments with elevated magnesium concentrations. Field studies of stromatolites in Salda Gölü (Lake) in southwestern Turkey [8-10] and the hydromagnesite playas of the Cariboo Plateau in British Columbia, Canada [11] have implicated cyanobacterial activity in the precipitation of magnesium carbonate, particularly hydromagnesite. However, direct experimental evidence connecting microbial activity to the precipitation of magnesium carbonates is lacking.

In this study, we examine the microbial life and carbonate mineralogy of benthic microbial mats in an alkaline wetland that is part of a hydromagnesite (Mg_5_(CO_3_)_4_(OH)_2_·4H_2_O) playa near Atlin, British Columbia, Canada. Our field observations indicate that nesquehonite (MgCO_3_·3H_2_O) forms as evaporative films on the wetland water surface whereas dypingite (Mg_5_(CO_3_)_4_(OH)_2_·5H_2_O) is localized in the benthic microbial mats. Field microcosm and laboratory precipitation experiments, which emulated natural conditions, were conducted to compare abiotic and biotic precipitation pathways. This includes a comparison of the resulting precipitate mineralogies, isotopic compositions, and the relative rates of precipitation. The results of this study have implications for the role of microbial communities in magnesium carbonate precipitation and demonstrate a strong connection between the biosphere and inorganic carbon pool.

### 2. Description of study area and sampling procedures

**Figure 1:**
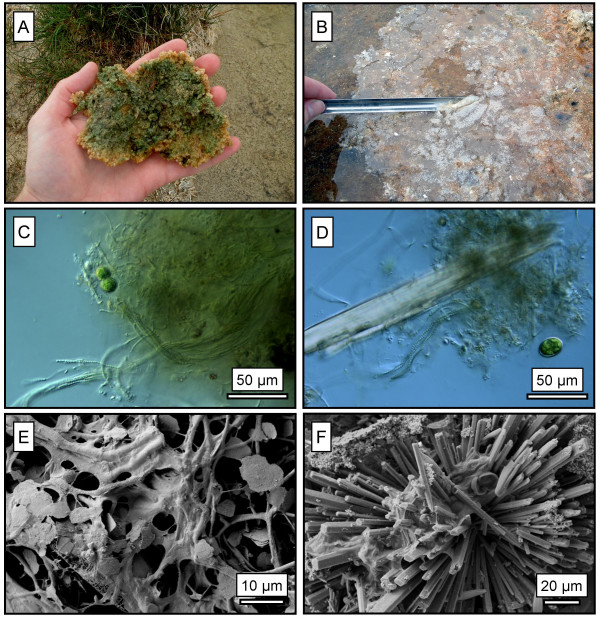
A: A photograph showing the underside of a benthic microbial mat found in the wetland. B: A film of nesquehonite on the wetland water surface. C: A light micrograph of the benthic microbial mats showing filamentous cyanobacteria, *Lyngbya *sp., and unicellular green algae. D: A second light micrograph of the microbial mats with a large crystal of nesquehonite that likely formed after sample collection. E: A SEM micrograph of flakey dypingite associated with filamentous cyanobacteria, which along with aragonite, constitutes the majority of the microbial mat material. F: Radial collection of nesquehonite crystals precipitated in a carbonate film on the water surface.

Hydromagnesite playas are located in topographic lows near Atlin, British Columbia. The bedrock in this region is ultramafic and represents a tectonically emplaced upper mantle section of oceanic lithosphere. The rocks are composed mainly of serpentinized harzburgite with variable carbonitized, and deformed harzburgite, and minor dunite lenses and pyroxenite veins [12]. Magnesium-rich groundwater is produced by the weathering of these rocks and may discharge into topographic lows. The playas have sharply defined boundaries and their surfaces are hummocky and slightly raised above the surrounding land. The hydromagnesite sediments of the playa have no bedding or other obvious sedimentary structures that show the preservation of microbial mats. However, raised parts have a fine scale (~0.5 cm) horizontal parting that is emphasized upon desiccation. The focus for field sampling was the wetland that exists as part of a playa located at 59°34'30" N, 133°41'60" W. The wetland has an area of approximately 0.5 ha and an average depth of ~0.3 m. There are no channelled inflows or outflows and therefore this wetland is considered to be groundwater fed. Within the wetland, there are a variety of microbial mats that are associated with carbonate sediments. Benthic microbial mats (Fig. 1A) are typically 2 cm in thickness, non-laminated and have a somewhat clotted internal fabric. These non-lithified, stratiform microbial mats are greenish brown on the surface and greener underneath.

Sampling at this site included the collection of water, sediment and microbial samples. To characterize the microbial communities, samples of microbial mats were collected using a 70% (v/v) ethanol-rinsed spatula, placed into sterile 15 ml plastic vials, and fixed in natural water using 2% (v/v) glutaraldehyde. These samples were examined and photographed using phase-contrast light microscopy (Zeiss Axioskop Mot 2 using a Retiga 1300 digital camera). To determine the mineralogy (by XRD) and stable isotope compositions, samples of mineralized microbial mat, mineral films on surface waters (Fig. 1B), and carbonate crusts on exposed surfaces were collected in 50 ml plastic vials. Additional samples of benthic microbial mats were collected to use as starting materials for culturing cyanobacteria biofilms to be used in laboratory test tube experiments.

Samples of wetland water and groundwater from a nearby well were filtered in the field (0.45 μm) into 125 ml polyethylene bottles for anion analyses. Sub-samples were acidified using concentrated trace-metal grade nitric acid (1.0% (v/v) final concentration) for cation analyses. Filtered waters were dispensed into 40 ml amber TraceClean vials with septa caps to be analyzed for stable carbon and oxygen isotope compositions. To obtain fresh groundwater, the well (59°34'40" N, 133°41'70" W) was flushed with a minimum of three volumes prior to sampling. The well is approximately 40 m from the wetland, ~33.5 to 35.0 m deep, and 15.2 cm wide. The water from the well was considered to be representative of the groundwater that feeds the wetland. It was assumed that the geochemical and microbial processes that occur in the wetland's surface waters have not altered the groundwater. Water and microbial samples were stored at 4°C until processing.

## 3. Methods

### 3.1 Field microcosm experiment

**Figure 2:**
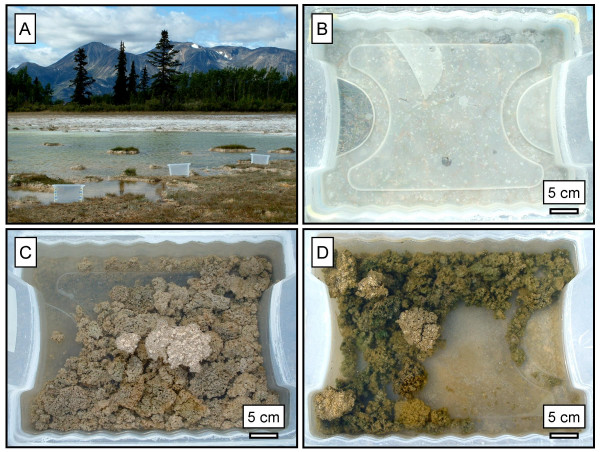
A: A photograph of the location and basic setup of the microcosm experiment. B-D: Overhead views of the microcosms on day 10 of the experiment. B: The abiotic microcosm showing an extensive crust of nesquehonite on the bottom of the container. C: The unamended mat and D: the amended eutrophic microcosms do not contain a nesquehonite crust. Also the mats in the eutrophic system are considerably greener than the unamended system indicating new microbial growth.

Microcosms were set-up in translucent polypropylene containers (~0.18 m^2^) to demonstrate carbonate precipitation under field conditions. Figure 2A shows the positioning of the three microcosms. The containers were not covered to best mimic the natural conditions of the wetland. Two microbial microcosms contained 15 L of unfiltered wetland water (depth ≈ 10 cm). Using nitrile gloves rinsed with 70% (v/v) ethanol and washed with wetland water, small portions (cm-scale thickness) of benthic mat were transferred to create a layer of natural microbial mat on the bottom of each of the two containers. Additional nutrients (0.5625 g K_2_HPO_4_, 22.5 g NaNO_3_; Allen's media concentration for 15 L [13]) were added to one of the microcosms to simulate eutrophic conditions at the beginning of the experiment (t = 0) and on day two to encourage microbial growth. An "abiotic" control contained 15 L of filtered (0.45 μm) wetland water. It was expected that the abiotic control would suffer minor contamination by airborne particles in the absence of a lid. This was considered acceptable given that minor contamination occurring after filtration wouldn't alter water composition in a significant manner relative to the microbial mats. At the end of the experiment, water from the control was sampled and examined using phase contrast light microscopy (Nikon Labophot^® ^light microscope) to assess the extent of contamination.

All three containers were placed in the wetland at a depth of approximately 10 cm below the water surface to provide similar environmental conditions (e.g., temperature and lighting) as the wetland. Water samples of each microcosm and the wetland were collected daily for a period of 10 days from August 11^th ^to 21^st^, 2005 for cation (15 ml) and anion (125 ml) analyses. In addition, pH and temperature were recorded daily. Grab samples of microbial mat and abiotic precipitate were obtained using a 70% (v/v) ethanol-rinsed spatula and placing them into sterile 50 ml plastic vials. Samples were analyzed for their mineralogical and isotopic (δ^13^C and δ^18^O) compositions.

### 3.2 Laboratory carbonate precipitation experiment

Enrichments of predominantly filamentous cyanobacteria were cultured from samples of sediment collected from the field site. Cyanobacteria were cultured from these sediments in 75 ml of Allen's media [13] in 125 ml Erlenmeyer flasks at room temperature (~20°C) and in the presence of sunlight. After sufficient growth, portions of biofilm were removed and cultured again. A total of three successive cultures for each enrichment were grown over a period of three months to produce carbonate-free filamentous cyanobacteria biomass to be used in precipitation experiments.

Precipitation experiments, similar to those conducted by Thompson and Ferris [2], were performed to evaluate the role cyanobacteria may play in precipitating magnesium carbonates under controlled laboratory conditions. Water samples included filtered (0.45 μm) groundwater from the nearby well and wetland water. Test tubes (16 × 150 mm) containing 10 ml of water were inoculated with biofilms (mm-scale thickness) of the cultured Atlin cyanobacteria. The wetland water experimental system received 15 mg NaNO_3 _and 0.375 mg K_2_HPO_4 _(Allen's media concentration for 10 ml; [13]) to promote microbial growth in the biotic system. Abiotic controls containing only filtered water were also prepared. Plastic push-caps were placed on top of each test tube to prevent contamination and minimize evaporation. To determine the effects of increasing pH value and hydroxyl concentration, a second abiotic control was prepared using the wetland water. Sodium hydroxide (0.5 M) was slowly added to 10 ml of filtered wetland water while monitoring pH until a macroscopic precipitate was observed.

Cultures and controls were incubated at room temperature in the presence of sunlight for six weeks. Biofilms were examined using phase-contrast microscopy to determine the presence of carbonate precipitates. While viewing under phase-contrast transmitted light microscope conditions, 'fizz' tests were performed using 10% (v/v) HCl to confirm the presence of carbonate minerals within the wet biofilms. This was done by preparing a conventional wet mount (~12 μl of culture) and placing a drop of acid (~25 μl) at the edge of the cover slip to be drawn under by capillary action to react with potential carbonate minerals within the biofilms [see Additional file 1]. As described later in the methods, water chemistry was analyzed for changes in [Mg^2+^], [Ca^2+^], [Si^4+^], and pH after the six-week incubation period. Samples of biofilms and secondary mineral precipitates were collected using a sterile spatula and examined using scanning electron microscopy (SEM). Precipitates were also collected and analyzed for their mineralogical and isotopic (δ^13^C and δ^18^O) compositions using micro X-ray diffraction and mass spectrometry, respectively.

### 3.3 Water chemistry

The pH values of natural and microcosm waters were measured with a HACH sension156 portable multiparameter meter. Alkalinity was determined in the field using the Gran function [14] for natural waters and PHREEQC was used to speciate the fluid chemistry [15]. In laboratory experiments, pH was measured using narrow range non-bleeding pH indicator strips (pH 6.5–10) from EM Science in order to avoid KCl contamination that may deleteriously affect bacterial growth. Cations were analyzed using a Perkin-Elmer 3300-DV ion-coupled plasma optical spectrometer (ICP-OES) and anions were analyzed using a Dionex IC-3000 ion chromatograph.

### 3.4 Scanning electron microscopy

Samples of benthic microbial mats, surface water carbonate films, and experimental biofilms and precipitates were examined. Each sample was placed onto a 12 mm carbon adhesive tab (EM Science) and excess water removed by capillary action using Whatman No. 1 filter paper and subsequently washed once with 100 μl of sterile dH_2_O using the same capillary method and air dried. The samples were prepared in this manner to prevent the formation of evaporative minerals that could be misinterpreted as primary precipitates. Traditional dehydration and critical point drying methods, which are used to preserve bacterial capsule [16], were avoided to prevent the dissolution of primary precipitates that may have formed in solution. Samples were gold-palladium coated using a Denton Vacuum Desk II gold-cathode sputter to reduce sample charging and analyzed using a ZEISS 1540 XB field emission gun – scanning electron microscope (FEG-SEM) at 3.0 kV. The SEM was equipped with an Oxford Instruments' INCAx-sight energy dispersive spectrophotometer (EDS) for elemental analysis using a voltage of 10 kV.

### 3.5 X-ray diffraction

Samples of benthic microbial mat, surface water carbonate films, abiotic microcosm precipitate, and abiotic control with NaOH from the laboratory experiment were analyzed by X-ray powder diffraction. The benthic microbial mat was gently pressed against Whatman No. 1 filter paper to remove water and therefore minimize the formation of evaporites. Mineral phases were identified at The University of British Columbia with X-ray powder diffraction data. Finely ground aliquots of sample were smear-mounted onto petrographic slides with anhydrous ethanol and allowed to dry at room temperature. XRD data for mineral identification were collected with a scanning step of 0.04° 2θ and counting time of 0.8 seconds per step on a Siemens θ–2θ D5000 diffractometer over a range of 3–80° 2θ. Mineral phases were identified with reference to the ICDD PDF4+ database using DIFFRAC^*plus *^EVA. The normal-focus Co-X-ray tube was operated at 35 kV and 40 mA.

Micro X-ray diffraction (μXRD) was employed for precipitates that had formed in biofilms in the laboratory experiments. Finely-ground aliquots of deionized H_2_O washed/air dried samples were placed onto a glass slide. The XRD data was collected using a Bruker D8 Discover diffractometer, which was operated with Cu*K*α radiation generated at 40 kV and 40 mA. A *K*α beam diameter of 500 μm was used to analyze several points in the sample for comprehensive mineral identification. Scans were collected over a range of 5–95° 2θ in a time interval of 8 minutes. Diffracted X-rays were detected using a General Area Diffraction Detector Systems (GADDS) 2D detector at a distance of ~15 cm from the sample. Constituent mineral phases were identified with reference to the ICDD database using DIFFRAC^*plus *^EVA.

### 3.6 Isotopic analysis

Carbonate samples from the field site, microcosm and laboratory experiments were analyzed for their carbon and oxygen isotopic compositions. Crushed samples of about 200 μg were analyzed using a ThermoFinnigan *DELTA*^*plus *^XL mass spectrometer with a Gasbench device at the Pacific Centre for Isotopic and Geochemical Research, The University of British Columbia. Samples were loaded in vials, flushed with helium, and dissolved in 99% phosphoric acid at 72°C for a minimum of one hour. The isotope compositions of the evolved headspace CO_2 _gas was measured in a flow of helium gas. The carbon and oxygen isotope compositions are reported in the conventional δ notation in per mil (‰) relative to the international standards Vienna Pee Dee Belemnite (VPDB) and Vienna Standard Mean Ocean Water (VSMOW), respectively. The external precision (1 sigma deviation) is <0.2‰ δ^18^O and <0.1‰ δ^13^C, as estimated from repeated analysis of in-house calcite standards, NBS-18, and NBS-19. At least three standards were analyzed for every eight samples. The ^18^O compositions of magnesium carbonate minerals were corrected for reaction with phosphoric acid using the fractionation values from Das Sharma *et al*. [17].

Carbon and oxygen isotopic analyses of the wetland and ground waters were performed at the G.G. Hatch Stable Isotope Laboratory, The University of Ottawa. Details regarding the carbon and oxygen isotope analyses of the water samples are provided by St-Jean [18] and Barth *et. al. *[19], respectively.

## 4. Results

### 4.1 Characterization of natural samples

The wetland within the playa had a pH of ~8.6 and high total alkalinity (4727 mg/L HCO_3_^-^). Mg concentration was as high as 780 ppm and the Mg:Ca ratio was typically greater than 50:1. For cations, the major elemental abundances were Mg^2+ ^>> Na^+ ^> Si^4+ ^> Ca^2+ ^> K^+^. Groundwater from the nearby well had the same relative elemental abundances, a pH of ~8.15, and alkalinity of ~2700 mg/L HCO_3_^-^. The ratio of Mg:Ca (65:1) was greater than in the wetland water. The stable isotopic compositions of the wetland (δ^13^C = 5.2‰, δ^18^O = -13.1‰) and groundwater (δ^13^C = -6.3‰, δ^18^O = -21.5‰) varied considerably. Refer to Table 1 for complete chemistry of these two waters.

**Table 1:** Chemistry of the Atlin wetland and ground waters.

Parameter (mg/L)	Groundwater	Wetland
pH	8.12	8.58
Alkalinity (mg/L HCO_3_)	2661	4727
δ^13^C (‰ VPDB)	-6.3	5.2
δ^18^O (‰ VSMOW)	-21.5	-13.1
Mg	456	778
Si	7.0	21.2
Na	14.6	75.1
Ca	6.9	14.3
K	4.9	9.3
Fe	0.04	0.1
Al	0.01	-
SO_4_	17.1	88.4
Cl	2.24	6.82
NO_3_	-	-
PO_4_	-	-
F	-	-

The evaporation of wetland water resulted in the formation of nesquehonite (MgCO_3_·3H_2_O) as sparse films (< mm-scale thickness) on the water surface in shallow areas, usually during relatively warm days. The nesquehonite in these thin films consisted of slender prisms that frequently radiate from a central point (Fig. 1F). A small amount of crystal aggregates (mm-scale) did settle in the water column and were incorporated in the benthic microbial mats. However, these nesquehonite film aggregates were sparse and did not appear to be a major contributor to the carbonate in the microbial mats. Nesquehonite (cm-scale thickness) was also identified as the crusts found on exposed surfaces adjacent to the water body.

**Figure 3:**
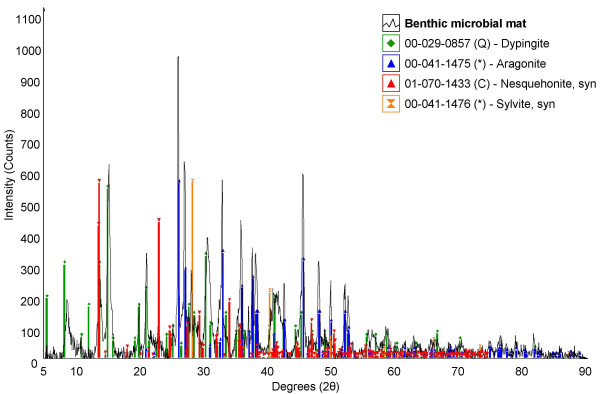
Powder X-ray diffraction pattern of minerals in the natural benthic mat. The plot is of X-ray beam intensity (background subtracted) versus 2θ and ICDD data for matching mineral phases are indicated by stick patterns.

The benthic microbial mat samples contained a variety of microorganisms but were dominated by *Lyngbya *sp., a filamentous cyanobacterium with a sheath and no basal cell (Fig. 1C). Figure 1D shows a portion of the biofilm with a large crystal of nesquehonite that likely precipitated after sample collection. Diatom species of *Navicula *and *Cyclotella *were also common in the wetland water. XRD analysis of the benthic mats found them to contain dypingite (Mg_5_(CO_3_)_4_(OH)_2_·5H_2_O) and aragonite with minor amounts of nesquehonite (MgCO_3_·3H_2_O) and sylvite (Fig. 3). The minor presence of nesquehonite may be the result of either abiotic precipitation in the wetland or by the evaporation of water from the mats after sampling as is certainly the case with sylvite (KCl). The dypingite was plate-like or flakey, and was typically associated with filamentous cyanobacteria (Fig. 1E). The occurrence of dypingite is restricted to the benthic microbial mats where cyanobacteria appear to create ideal conditions for carbonate formation as discussed later.

### 4.2 Field microcosm experiment

**Figure 4:**
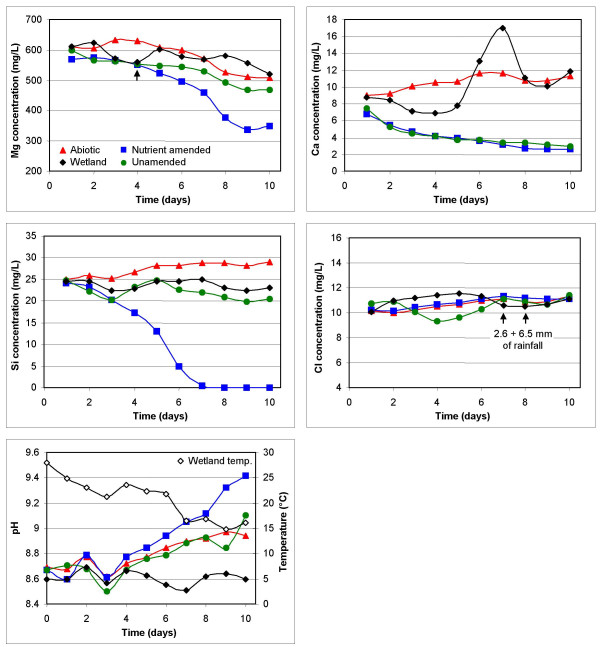
Graphs showing changes in [Mg^2+^], [Ca^2+^], [Si^4+^], [Cl^-^], and pH in the wetland (black diamonds), abiotic control (red triangles), unamended (green circles), and amended microbial mat (blue squares) microcosms with time. The data is presented as a 2-day moving average. The arrow on the Mg-graph indicates the day when new microbial growth was seen as greening of the microbial mats in the amended microcosm. The arrows placed on the Cl-graph show the occurrences of two rainfall events on days 7 (2.6 mm) and 8 (6.5 mm). The pH graph also displays wetland temperature (black open diamonds), which matches microcosm temperatures.

The microcosm experiment was designed to compare abiotic and biotic precipitation of magnesium carbonates under field conditions. Figure 4 shows the changes in [Mg^2+^], [Ca^2+^], [Si^4+^], [Cl^-^], and pH of the wetland and three microcosms as well as the wetland's temperature at mid-afternoon during the times of sampling. The wetland is a very small water body and therefore its temperature is regulated by the surrounding air. As well, microcosm temperatures matched those of the wetland. Minor rainfall events occurred on days seven (2.6 mm) and eight (6.5 mm).

The wetland's pH (avg. = 8.61), calcium (avg. = 10.2 mg/L), silicon (avg. = 23.7 mg/L) and chloride (avg. = 10.9 mg/L) concentrations fluctuated, but did not show any discernible trends. Magnesium concentration did decrease from 612 to 519 mg/L over the course of the experiment. In the abiotic system, clusters of crystals were noticeable on the water surface from day two and by day 10 a mineral crust (~0.5 cm thickness) covered the entire bottom of the container (Fig. 2B). The crust was made of nesquehonite (MgCO_3_·3H_2_O; [20]) and minor dypingite. The dypingite may have resulted from decomposition of nesquehonite between the time of collection and analysis. The examination of > 10 fields of view of the abiotic control using phase contrast light microscopy did not reveal any bacteria, demonstrating that contamination was < 10^3^/ml, or less than 1 in 10^6 ^relative to the biotic systems. Over the course of the 10 days the pH value increased from 8.69 to 8.94 while Mg concentration decreased from 611 to 509 mg/L and the Ca concentration increased from 8.95 to 11.3 mg/L.

In the two microbial microcosms, minor amounts of crystals were visible on the water surface. However, a mineral crust, like the one formed in the abiotic system, did not develop in either system (Fig. 2C–D). In the unamended microcosm the pH value increased from 8.67 to 9.1 and the Mg and Ca concentrations decreased from 600 to 467 mg/L and 7.44 to 2.93 mg/L, respectively. The nutrient amendment to the eutrophic system resulted in the formation of new biomass (seen as greening of the mats) that was recognizable after four days. At the end of the experiment, cyanobacteria had begun to colonize the bottom of the container. Magnesium and calcium concentrations decreased from 569 to 349 mg/L and 6.79 to 2.67 mg/L, respectively. The pH value (9.42) was notably higher than the unamended microbial system. Even though water compositions indicate that Mg and Ca were removed from the waters it was not possible to determine the mineralogy of any new precipitates by XRD due to the overwhelming presence of pre-existing carbonate. However, it was determined that the mats contained greater amounts of nesquehonite than in the natural samples. Nesquehonite was recognised precipitating on the top surface of mats that occasionally floated to the surface due to the trapping of oxygen bubbles. As expected, this was more common in the nutrient amended microcosm.

In the three microcosms, chloride concentrations increased by an average of 9% due to evaporation. Also noteworthy were changes in silicon concentrations, which increased in the abiotic system due to evapoconcentration and decreased in the microbial systems due to the growth of diatoms. The proliferation of diatoms under eutrophic conditions caused a nearly complete depletion of silicon (initially 24.3 mg/L).

### 4.3 Laboratory carbonate precipitation experiment

**Figure 5:**
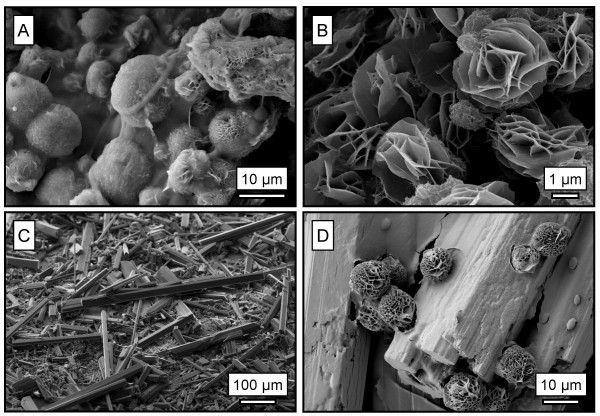
SEM micrographs of experimental samples. A: Rosettes of dypingite, covered with extracellular organic material, precipitated from groundwater in association with a biofilm of cyanobacteria. B: Rosette-like dypingite precipitated from Atlin wetland in the presence of cyanobacterial consortium. C: Slender prismatic crystals of nesquehonite precipitated from Atlin wetland water in the abiotic system. D: Dypingite rosettes on prismatic crystals of nesquehonite precipitated from wetland water with the addition of NaOH.

**Figure 6:**
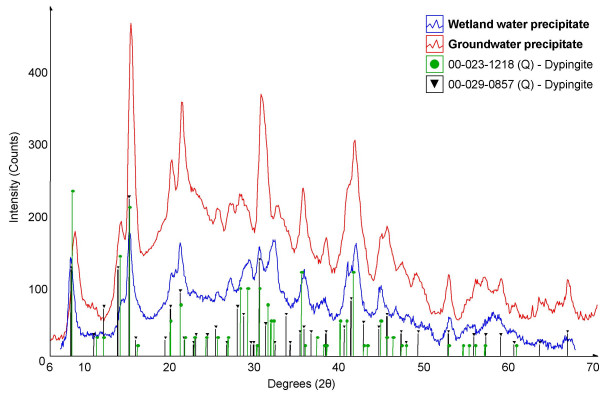
Micro X-ray diffraction pattern of precipitates formed in association with experimental biofilms using wetland and ground waters from the Atlin site. The plot is of background subtracted X-ray intensity versus 2θ and matching mineral phases from the ICDD database shown by stick patterns.

The laboratory experiment compared abiotic and biotic carbonate precipitation under controlled conditions and allowed for the characterization of newly formed precipitates. Both groundwater and wetland water supported the growth of extensive filamentous biofilms (~1 mm thick) that coated the inside of the test tubes. The biofilms were seen encrusted with spherical, transparent mineral precipitates when viewed using phase-contrast light microscopy. These precipitates produced a gaseous phase (presumably CO_2_) when exposed to dilute hydrochloric acid suggesting a carbonate phase. When examined using SEM, rosettes with minor amounts of flakey globular aggregates were seen in the biofilm that formed from the groundwater (Fig. 5A). Rosette-like forms were also observed in the biotic system containing wetland water (Fig. 5B). EDS analyses of these precipitates showed they contained Mg, C, and O thereby identifying them as magnesium carbonates. Rosettes (8–12 μm in diameter) precipitated from the groundwater were typically twice the size of those precipitated from the wetland water (4–5 μm diameter). μXRD analysis (Fig. 6) of these precipitates determined that the predominate mineral phase was dypingite (Mg_5_(CO_3_)_4_(OH)_2_·5H_2_O). No nesquehonite was detected in either biological system.

In the abiotic control containing wetland water, single prismatic crystals and radial aggregates of crystals were observed (Fig. 5C). These precipitates were identified as nesquehonite (MgCO_3_·3H_2_O) by their characteristic crystal habit, XRD pattern, and EDS analysis of their composition (Mg, C, O). Precipitation did not occur in the abiotic control containing groundwater, as indicated by the increase in magnesium concentration due to evaporation (Table 2). The drop wise addition of 0.1 M NaOH to wetland water while mixing resulted in the formation of a milky suspension at pH 10.30. The final precipitate was found to be composed of approximately equal parts of nesquehonite, dypingite, and an unknown Mg-carbonate (Fig. 5D).

**Table 2:** Basic chemistry of the natural waters used in precipitation experiments and the resulting experimental mineral precipitates.

Sample	Initial chemistry		Final chemistry (6 weeks)				
	pH	Cation (mg/L)	System	pH	Cation (mg/L)	Percent change	Precipitate mineralogy
Ground water	8.12	Mg – 340	abiotic	9.2	Mg – 352	+3.5%	no precipitate
		Ca – 4.00			Ca – 4.48	+12%	
		Si – 4.55			Si – 5.36	+18%	
			biotic	9.6	Mg – 134	-61%	dypingite
					Ca – 2.45	-39%	
					Si – 1.73	-62%	
Wetland water	8.58	Mg – 780	abiotic	9.0	Mg – 457	-41%	nesquehonite
		Ca – 11.1			Ca – 6.25	-44%	
		Si – 18.9			Si – 19.9	+5.3%	
			biotic	9.5	Mg – 177	-77%	dypingite
					Ca – 1.99	-82%	
					Si – 0.16	-99%	
w/ NaOH			abiotic	10.3	-		nesquehonite w/ dypingite

Comparing water compositions in the abiotic versus biotic experiments (Table 2), the Mg and Ca concentrations were found to decrease significantly more in the biotic systems; indicating a greater amount of carbonate precipitation in the presence of cyanobacteria. The pH was also higher in the biotic systems due to photosynthesis [2].

## 5. Discussion

### 5.1 Precipitation experiments

The field microcosm experiment contrasted carbonate precipitation under three separate sets of conditions: purely abiotic, unamended nutrient and amended (eutrophic) nutrient conditions. Magnesium concentrations in the microcosm waters decreased by 16.7%, 22.2% and 38.7% in these three microcosms, respectively. These decreases in [Mg^2+^] would be lower if not for evapoconcentration as noted by the increases in chloride concentrations. The decline in dissolved magnesium in all systems is a clear indicator of Mg-carbonate precipitation. However, the greater decreases of Mg and Ca in the biotic microcosms suggest that carbonates are forming in the presence of cyanobacteria at lower concentrations than that needed for purely abiotic precipitation to occur. Furthermore, the addition of nutrients (N and P) was successful in elevating microbial activity resulting in greater depletion of Mg and more carbonate precipitation. Therefore, an increase in microbial activity results in an increase of the carbonate precipitation rate in the wetland environment. In the abiotic microcosm, nesquehonite formed over time due to evaporation of wetland water. Mineral identification of new precipitates in the microbial microcosms was not possible due to the presence of pre-existing carbonates. However, mineral precipitation occurred within the microbial mats and mineral crusts similar to the one formed in the abiotic control did not form in either biotic system even where mats had shifted exposing the bottom of the container. This demonstrates that carbonates in the benthic mats are not simply precipitates that form and settle from overlying waters. The greater amount of nesquehonite detected in the eutrophic mats can be attributed to evaporation from floating segments of mat. Similarly, McLean *et al*. [21] formed nesquehonite on the surface of a biofilm when its host water was allowed to evaporate from the system. Floating segments of mat do not appear in the wetland, but cm-scale crusts of nesquehonite are present along the shorelines where benthic mats can become exposed.

The results of the laboratory experiments suggest that cyanobacteria promote carbonate formation at a faster rate and, more importantly, of a different mineralogy than the abiotic systems. In association with experimental biofilms, dypingite was the primary precipitate. Dypingite is a rare mineral that was first described by Raade [22] as an alteration product of serpentine. Rosettes precipitated from the wetland water were smaller than those formed from the groundwater likely as a result of higher Mg concentration and hence more nucleation sites. Hydromagnesite and dypingite are remarkably similar in composition and both may form rosette morphologies [22,23] Very similar habits of hydromagnesite have been observed in natural stromatolite samples from Salda Gölü, Turkey [8-10]. SEM images by Braithwaite and Zedef [9] showed there was little evidence of direct crystallization on organic surfaces. However, in natural settings, microbial growth and decay continually over as little as several years can alter the original microbe-mineral configuration making it difficult to identify a direct association between extant microbial life and precipitates. An unmistakably direct association between cyanobacteria and mineral precipitates can be seen by examining an active system as in this study. For example, the dypingite rosettes shown in Fig. 5A are coated in extracellular organic matter. The abiotic control with wetland water precipitated only nesquehonite just as the abiotic control in the microcosm experiment. In the pH adjusted abiotic control, the addition of NaOH (final pH 10.3) caused the precipitation of dypingite rosettes similar to those associated with the biofilms. However, nesquehonite continued to be the dominant precipitate favoured under abiotic conditions, even at higher pH values. This suggests that pH within the experimental biofilms must have been at least one pH unit greater than the final bulk solution pH (9.5 and 9.6 for the wetland and ground waters, respectfully). Furthermore, it strongly indicates that the hydroxyl ions produced during photosynthesis [2] promote precipitation of dypingite.

### 5.2 Stable carbon and oxygen isotopes

**Figure 7:**
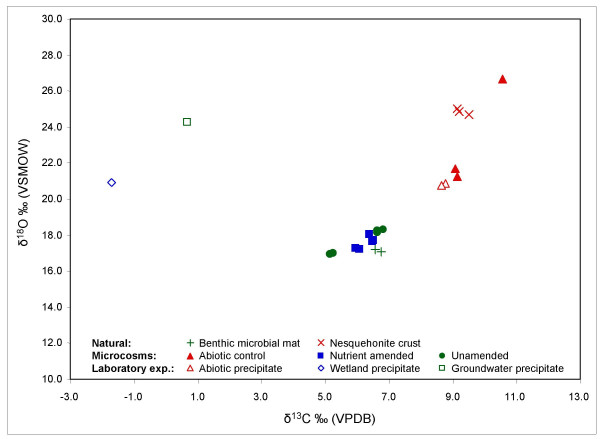
Isotope compositions (δ^18^O vs. δ^13^C) of natural samples of benthic microbial mat and nesquehonite crusts, as well as precipitates from the field microcosm and laboratory precipitation experiments.

Carbon and oxygen isotopic compositions of natural benthic microbial mat and nesquehonite crust as well as microbially and abiotically formed precipitates from the microcosm and laboratory experiments are shown in Fig. 7. Enrichment in both ^13^C and ^18^O of the wetland water (δ^13^C = 5.2‰, δ^18^O = -13.1‰) relative to the groundwater (δ^13^C = -6.3‰, δ^18^O = -21.5‰) can be attributed to degassing of CO_2 _and evaporation of water depleted of heavy isotopes.

In the natural environment, significant isotopic differences exist between abiotically formed nesquehonite (δ^13^C avg. = 9.3‰, δ^18^O avg. = 24.9‰) and carbonates present in benthic microbial mat (δ^13^C avg. = 6.7‰, δ^18^O avg. = 17.2‰). A modest ^13^C enrichment of 1.5‰ of the carbonate in the benthic mats compared to DIC of the wetland water can be attributed to fractionation during precipitation and possibly preferential removal of isotopically light bicarbonate during photosynthesis. Pentecost and Spiro [24] calculated that a removal of 17% of total DIC in the interstitial water of a *Rivularia *sp. colony via photosynthesis would increase DIC ^13^C by 2‰. There will also be a Rayleigh distillation effect in the somewhat isolated, interstitial water of the benthic mats whereby the bicarbonate reservoir becomes progressively depleted in ^18^O and ^13^C as carbonate precipitation progresses. Respiration and Rayleigh distillation occurring in microbial mats will tend to produce carbonates that are depleted in ^13^C and ^18^O relative to those precipitated abiotically. This effect is evident when comparing the δ-values of benthic mat carbonate to the nesquehonite crusts. Both samples precipitated from the same water, but the benthic mat carbonate is depleted in ^13^C and ^18^O by an average of 2.6‰ and 7.7‰, respectively. The relative importance of photosynthesis and respiration in the microbial mats will vary with seasonal conditions [24]. The ^18^O enrichment in nesquehonite, which forms as an evaporite, can be attributed to fractionation during evaporation.

In the microbial microcosms, the overwhelming presence of pre-existing carbonate likely masked any isotopic signal from newly precipitated carbonates. Consequently, carbon and oxygen isotope values of the unamended mats (δ^13^C avg. = 6.1‰, δ^18^O avg. = 17.7‰) and amended mats (δ^13^C = 6.3‰, δ^18^O = 17.6‰) were very similar to natural benthic mat (δ^13^C avg. = 6.7‰, δ^18^O avg. = 17.2‰). The slight depletion of ^13^C and enrichment in ^18^O relative to the natural benthic mats can be attributed to Rayleigh fractionation of the bicarbonate reservoir and evaporation of the water in the small reservoir, respectively. The isotopic compositions of the precipitates in the abiotic microcosm varied more than those in the microbial systems. However, the nesquehonite crust formed in the abiotic microcosm (δ^13^C avg. = 9.6‰, δ^18^O avg. = 23.2‰) had similar carbon and oxygen isotope values as the natural nesquehonite crust (δ^13^C avg. = 9.3‰, δ^18^O avg. = 24.9‰). Isotope data from this experiment suggest that the microcosms were successful in simulating conditions for both biotic and abiotic precipitation similar to those in the wetland.

In the laboratory experiment, precipitates from wetland water (δ^13^C = -1.7‰, δ^18^O = 20.9‰) and groundwater (δ^13^C = 0.7‰, δ^18^O = 24.3‰) that formed in association with cyanobacteria were depleted in ^13^C and enriched in ^18^O relative to natural benthic microbial mat carbonates (δ^13^C avg. = 6.7‰, δ^18^O avg. = 17.2‰). These differences in δ^13^C and δ^18^O values are attributed to very strong Rayleigh fractionation and minimal evaporation occurring over the six week experiment in these 'closed' systems (test tubes). The dypingite that formed from wetland water had depleted δ^13^C and δ^18^O values relative to the dypingite precipitated from groundwater. Greater Mg-carbonate precipitation and possibly greater respiration in the wetland water test tube would result in a stronger Rayleigh distillation effect and a lower δ^13^C value relative to the groundwater precipitate. Less evaporation in the test tube with wetland water may also account for the heavier oxygen isotope values.

### 5.3 Carbonate precipitation pathways

In this study, the presence of both an abiotic and biotic precipitation pathway offers insight into the processes involved in cyanobacterial precipitation of magnesium carbonates. This is accomplished by comparing the conditions that form nesquehonite (abiotic) and dypingite/hydromagnesite (biotic) in this environment. Groundwater from the well is near saturation with respect to hydromagnesite and becomes supersaturated upon degassing and evaporation after discharging into the wetland. Thermodynamic data does not exist for dypingite. However, given the similarity in chemical formulas, hydromagnesite is likely to be a close proxy for dypingite. Upon further evaporation, wetland water can become supersaturated with respect to nesquehonite. This can be seen in shallow near shore sections of the wetland where evaporative films of nesquehonite form on the water surface. Documented occurrences of nesquehonite indicates that it forms as an evaporative mineral or as an alteration product of carbonates, serpentines, volcanic breccias, meteorites and synthetic materials such as bricks and mortar [20]. Although hydromagnesite has a lower solubility than nesquehonite [25], it appears to be kinetically inhibited in forming by evaporation of the wetland water.

Cyanobacteria have been shown to overcome kinetic barriers and induce carbonate precipitation. Comparing the chemical formulas of nesquehonite (MgCO_3_·3H_2_O) and dypingite (Mg_5_(CO_3_)_4_(OH)_2_·5H_2_O) indicates that dypingite will generally form at a higher pH value, and lower CO_2 _and H_2_O activities than nesquehonite. The dominate cyanobacterium in the benthic mats, *Lyngbya *sp., has been implicated in carbonate formation of travertine deposits [26]. Pentecost [26] studied the spatial arrangement of *Lyngbya *on travertine crusts and described microniches as spaces in the community structure of filamentous cyanobacteria. The interstitial water in these microniches can be dramatically altered, relative to ambient water, by biological processes, which produce geochemical conditions that favour carbonate precipitation. Photosynthesis causes the alkalization of this microenvironment by removing bicarbonate and generating hydroxyl anions (Eqn. 1; [2,26]).

(1) HCO_3_^- ^+ H_2_O + *hv *→ CH_2_O + OH^- ^+ O_2_↑

The increase in pH value forces Equation 2 forward thereby increasing [CO_3_^2-^] activity.

(2) HCO_3_^- ^+ OH^- ^→ H_2_O + CO_3_^2- ^

High hydroxyl activity will also promote the formation of both dypingite and hydromagnesite. Nothdurft *et al*. [27] suggest that production of ammonia by heterotrophic bacteria, which are associated with cyanobacteria, can create an excess of OH^-^. This may lead to precipitation of brucite (MgOH_2_) at higher pH values.

Microbial cell walls provide an ideal surface for mineral nucleation with large numbers of regularly-spaced, chemically-identical nucleation sites (e.g., carboxyl groups and amino functional groups, Eqn. 3). They also possess the ability to concentrate cations, such as Mg^2+ ^and Ca^2+ ^from solution due to the net-negative surface charge that occurs on most bacteria [28]. Adsorbed magnesium ions will also act as salt bridges for attracting the counter ions, bicarbonate and carbonate [9].

(3) R·COO^- ^+ Mg^2+ ^→ R·COOMg^+ ^

Obst *et al*. [29] performed cation adsorption experiments under different concentrations of Ba^2+^, Ca^2+^, Sr^2+^, Mg^2+ ^(0 to 22 mmol) using live cells of the freshwater cyanobacterium, *Synechococcus leopoliensis*. The authors found that the relative effectiveness of each divalent cation at reducing the net-negative surface charge of the bacterium was Ba^2+ ^> Ca^2+ ^> Sr^2+ ^> Mg^2+^. They suggested that the very strong hydration of Mg^2+ ^ions may be reducing its ability to adsorb onto the cell membrane. In fact, one of the key barriers in forming non-hydrated magnesium carbonates (magnesite and dolomite) is the strong hydration of Mg^2+ ^that has a hydration energy of 1926 kJ/mol compared to 1579 kJ/mol for Ca^2+ ^[30,31]. Six water molecules in octahedral coordination surround the Mg^2+ ^ion in a rigid first solvent shell [32]. For comparison, the exchange rate of water in the hydration shell of Ca^2+ ^ions is ~1000-fold faster than for Mg^2+ ^ions [33]. Organic ligands within a biofilm or on the cell membrane may cause partial dehydration of Mg^2+ ^ions and allow for further association with carbonate anions [Eqn. 3; [34,35]].

The presence of adsorbed Mg^2+ ^ions and possibly their partial dehydration on cellular surfaces (Eqn. 3) with elevated CO_3_^2- ^and OH^- ^activities in the immediate vicinity of the cell wall results in conditions of supersaturation with respect to dypingite (Eqn. 4). Purely abiotic processes appear to form nesquehonite (39.1 wt. % H_2_O; Eqn. 5), a much more hydrated magnesium carbonate compared to dypingite (22.3 wt. % H_2_O).

(4) 5Mg^2+ ^+ 4CO_3_^2- ^+ 2OH^- ^+ 5H_2_O → Mg_5_(CO_3_)_4_(OH)_2_·5H_2_O

(5) Mg^2+ ^+ CO_3_^2- ^+ 3H_2_O → Mg(CO_3_)·3H_2_O

The biologically mediated formation of dypingite in this system likely serves as a precursor for the formation of hydromagnesite via dehydration (Eqn. 6). The wetting and drying cycles of the playa provides the appropriate environment for this dehydration reaction to occur.

(6) Mg_5_(CO_3_)_4_(OH)_2_·5H_2_O → Mg_5_(CO_3_)_4_(OH)_2_·4H_2_O + H_2_O

The identification of the natural carbonates in the Atlin wetland and the role of cyanobacteria dominated biofilms in the formation of dypingite in the experimental systems (versus the minor amounts of nesquehonite formed through abiotic processes) indicate the importance of the biosphere to the formation of this deposit. Filamentous cyanobacteria catalyze the precipitation of magnesium carbonate minerals faster than abiotic processes and produce mineral phases that differ from those formed abiotically.

## 6. Conclusion

The geochemical, mineralogical and microbiological examination of the natural Atlin wetland and experiments document the importance of cyanobacteria in promoting the formation of magnesium carbonate minerals. In this alkaline wetland, evaporation produces nesquehonite films on the water surface and crusts on exposed surfaces whereas dypingite is found in benthic microbial mats. The presence of biotic and abiotic precipitation pathways allow for a comparison of the conditions that lead to the precipitation of these two minerals. Field and laboratory experiments successfully emulated natural conditions and demonstrated that microbial mats and biofilms dominated by cyanobacteria can precipitate carbonates at faster rates than abiotic controls. These biofilms produced carbonates that were mineralogically and isotopically different than carbonates formed in the abiotic systems.

## 7. Authors' contributions

IP designed and conducted the experiments, carried out microscopy, and drafted the manuscript. SW conducted the mineralogical and isotopic analyses. JT performed geochemical modeling and contributed water chemical analyses. GD contributed to study design and oversaw data analysis. GS conceived of the study and advised on all aspects of this research. All authors participated in fieldwork and the editing process. As well, all authors read and approved of the final manuscript.

## Supplementary Material

Additional file 1 Light microscopy acid-carbonate technique. The additional data file (LM acid-carbonate technique.wmv) is a light microscopy video demonstrating a simple acid test for determining the presence of carbonate within hydrated biofilms. By placing a small drop of dilute HCl on the edge of the cover slip the acid is drawn under and will react with any carbonate minerals present. The video shows extensive degassing of carbon dioxide being released from a biofilm that had no recognizable carbonate minerals. This technique is most useful for determining the presence of minuscule amounts of carbonate within a biofilm. For example, even sub-micron size particles of carbonate produced enough CO_2 _to form a bubble that was visible using light microscopy. This is an inexpensive and simple technique that allows one to screen samples prior to using more advanced techniques such as scanning or transmission electron microscopy. Click here for file

## References

[B1] Riding R Microbial carbonates: The geological record of calcified bacterial-algal mats and biofilms Sedimentology 2000 47 179 214 10.1046/j.1365-3091.2000.00003.x

[B2] Thompson JB Ferris FG Cyanobacterial precipitation of gypsum, calcite, and magnesite from natural alkaline lake water Geology 1990 18 995 998 10.1130/0091-7613(1990)018<0995:CPOGCA>2.3.CO;2

[B3] Dittrich M Müller B Mavrocordatos D Wehrli B Induced calcite precipitation by cyanobacterium Synechococcus Acta Hydrochim Hydrobiol 2003 31 162 169 10.1002/aheh.200300486

[B4] Yates KK Robbins LL Production of carbonate sediments by a unicellular green alga Am Mineral 1998 83 1503 1509

[B5] van Lith Y Warthmann R Vasconcelos C McKenzie JA Microbial fossilization in carbonate sediments: a result of the bacterial surface involvement in dolomite precipitation Sedimentology 2003 50 237 245 10.1046/j.1365-3091.2003.00550.x

[B6] van Lith Y Warthmann R Vasconcelos C McKenzie JA Sulphate-reducing bacteria induce low-temperature Ca-dolomite and high Mg-calcite formation Geobiology 2003 1 71 79 10.1046/j.1472-4669.2003.00003.x

[B7] Roberts JA Bennett PC González LA Macpherson GL Milliken KL Microbial precipitation of dolomite in methanogenic groundwater Geology 2004 32 277 280 10.1130/G20246.2

[B8] Braithwaite CJR Zedef V Living hydromagnesite stromatolites from Turkey Sed Geol 1994 92 1 5 10.1016/0037-0738(94)90051-5

[B9] Braithwaite CJR Zedef V Hydromagnesite stromatolites and sediments in an alkaline lake, Salda Gölü, Turkey J Sed Res 1996 66 991 1002

[B10] Russell MJ Ingham JK Zedef V Maktav D Sunar F Hall AJ Fallick AE Search for signs of ancient life on Mars: Expectations from hydromagnesite microbialites, Salda Lake, Turkey J Geol Soc London 1999 156 869 888 10.1144/gsjgs.156.5.0869

[B11] Renaut RW Morphology, distribution, and preservation potential of microbial mats in the hydromagnesite-magnesite playas of the Cariboo Plateau, British-Columbia, Canada Hydrobiologia 1993 267 75 98 10.1007/BF00018792

[B12] Hansen LD Dipple GM Gordon TM Kellett DA Carbonated serpentinite (listwanite) at Atlin, British Columbia: A geological analogue to carbon dioxide sequestration Can Mineral 2005 43 225 239 10.2113/gscanmin.43.1.225

[B13] Allen MM Simple conditions for growth of unicellular blue-green algae on plates J Phycol 1968 4 1 4 10.1111/j.1529-8817.1968.tb04667.x 27067764

[B14] Lahav O Morgan BE Loewenthal RE Measurement of pH, alkalinity and acidity in ultra-soft waters Water SA 2001 27 423 431

[B15] Parkhurst DL Appelo CAJ User's guide to PHREEQC (version 2) – A computer program for speciation, batch reaction, one-dimensional transport, and inverse geochemical calculations 1999 U.S. Geological Survey Water-Resources Investigations Report 99 4259

[B16] Moser DP Onstott TC Fredrickson JK Brockman FJ Takai K Balkwill DL Drake G Pfiffner S White DC Baker BJ Pratt LM Fong J Sherwood Lollar B Slater G Phelps TJ Spoelstra N DeFlaun M Southam G Welty AT Hoek J Evolution of Microbial Community Structure and Geochemistry in an Ultradeep South African Gold Mine Borehole Geomicrobiol J 2003 20 517 548 10.1080/713851170

[B17] Das Sharma S Patil DJ Gopalan K Temperature dependence of oxygen isotope fractionation of CO_2 _from magnesite-phosphoric acid reaction Geochim Cosmochim Acta 2002 66 589 593 10.1016/S0016-7037(01)00833-X

[B18] St-Jean G Automated quantitative and isotopic (^13^C) analysis of dissolved inorganic carbon and dissolved organic carbon in continuous-flow using a total organic carbon analyser Rapid Commun Mass Spectrom 2003 17 419 428 10.1002/rcm.926 12590390

[B19] Barth JAC Tait A Bolshaw M Automated analyses of ^18^O/^16^O ratios in dissolved oxygen from 12-mL water samples Limnol Oceanogr: Methods 2004 2 35 41

[B20] Kloprogge JT Martens WN Nothdurft L Duong LV Webb GE Low temperature synthesis and characterization of nesquehonite J Mater Sci Lett 2003 22 825 829 10.1023/A:1023916326626

[B21] McLean RJC Jamieson HE Cullimore DR Formation of nesquehonite and other minerals as a consequence of biofilm dehydration World J Microb Biot 1997 13 25 28 10.1007/BF02770803

[B22] Raade G Dypingite, a new hydrous basic carbonate of magnesium, from Norway Am Mineral 1970 55 1457 1465

[B23] Canterford JH Tsambourakis G Some observations on the properties of dypingite, Mg_5_(CO_3_)_4_(OH)_2 _·5H_2_O, and related minerals Mineral Mag 1984 48 437 442 10.1180/minmag.1984.048.348.15

[B24] Pentecost A Spiro B Stable carbon and oxygen isotope composition of calcites associated with modern freshwater cyanobacteria and algae Geomicrobiol J 1990 8 17 26 10.1080/01490459009377875

[B25] Königsberger E Königsberger L Gamsjäger H Low-temperature thermodynamic model for the system Na_2_CO_3_-MgCO_3_-CaCO_3_-H_2_O Geochim Cosmochim Acta 1999 63 3105 3119 10.1016/S0016-7037(99)00238-0

[B26] Pentecost A Significance of the biomineralizing microniche in a Lyngbya (cyanobacterium) travertine Geomicrobiol J 1995 13 213 222 10.1080/01490459509378022

[B27] Nothdurft LD Webb GE Buster NA Holmes CW Sorauf JE Kloprogge JT Brucite microbialites in living coral skeletons: Indicators of extreme microenvironments in shallow-marine settings Geology 2005 33 169 172 10.1130/G20932.1

[B28] Schultze-Lam S Fortin D Davis BS Beveridge TJ Mineralization of bacterial surfaces Chem Geol 1996 132 171 181 10.1016/S0009-2541(96)00053-8

[B29] Obst M Dittrich M Kuehn H Calcium adsorption and changes of the surface microtopography of cyanobacteria studied by AFM, CFM, and TEM with respect to biogenic calcite nucleation Geochem Geophy Geosy 2006 7 Q06011 doi:101029/2005GC001172. 10.1029/2005GC001172

[B30] Slaughter M Hill RJ The influence of organic matter in organogenic dolomitization J Sed Petrol 1991 61 296 303

[B31] Wright DT Wacey D Precipitation of dolomite using sulphate-reducing bacteria from the Coorong Region, South Australia: Significance and implications Sedimentology 2005 52 987 1008 10.1111/j.1365-3091.2005.00732.x

[B32] Kluge S Weston J Can a hydroxide ligand trigger a change in the coordination number of magnesium ions in biological systems Biochemistry 2005 44 4877 4885 10.1021/bi047454j 15779914

[B33] Fenter P Zhang Z Park C Sturchio NC Hu XM Higgins SR Structure and reactivity of dolomite (104)-water interface: New insights into the dolomite problem Geochim Cosmochim Acta 2007 71 566 579 10.1016/j.gca.2006.10.006

[B34] Dudev T Cowan JA Lim C Competitive binding in magnesium coordination chemistry: Water versus ligands of biological interest J Am Chem Soc 1999 121 7665 7673 10.1021/ja984470t

[B35] Pokrovsky OS Schott J Kinetics and mechanism of dolomite dissolution in neutral to alkaline solutions revisited Am J Sci 2001 301 597 626 http://www.ajsonline.org/cgi/content/abstract/301/7/597 10.2475/ajs.301.7.597

